# (+)-Dehydrovomifoliol Alleviates Oleic Acid-Induced Lipid Accumulation in HepG2 Cells *via* the PPARα–FGF21 Pathway

**DOI:** 10.3389/fphar.2021.750147

**Published:** 2021-11-19

**Authors:** Yiyuan Xi, Jujia Zheng, Wei Xie, Xiangwei Xu, Namki Cho, Xudong Zhou, Xiaomin Yu

**Affiliations:** ^1^ School of Pharmaceutical Sciences, Wenzhou Medical University, Wenzhou, China; ^2^ Research Institute of Pharmaceutical Sciences, College of Pharmacy, Chonnam National University, Gwangju, Korea; ^3^ Pharmacy Department, Yongkang First People’s Hospital, Jinhua, China; ^4^ TCM and Ethnomedicine Innovation and Development International Laboratory, Innovative Materia Medica Research Institute, School of Pharmacy, Hunan University of Chinese Medicine, Changsha, China

**Keywords:** artemisia frigida, non-alcoholic fatty liver disease, peroxisome proliferator-activated receptorα, lipid accumulation, terpene

## Abstract

An overload of hepatic fatty acids, such as oleic acid is a key trigger of non-alcoholic fatty liver disease (NAFLD). Here, we investigated whether *Artemisia frigida*, a valuable traditional medicine used to treat various diseases, could mitigate OA-induced lipid accumulation in HepG2 cells. Then, to identify the active substances in *A. frigida*, a phytochemistry investigation was conducted using a bioassay-guided isolation method. Consequently, one terpene (**1**) and one flavone (**2**) were identified. Compound **1** ((+)-dehydrovomifoliol) exhibited potent effects against lipid accumulation in OA-induced HepG2 cells, without causing cyto-toxicity. Notably, treatment with (+)-dehydrovomifoliol decreased the expression levels of three genes related to lipogenesis (*SREBP1, ACC,* and *FASN*) and increased those of three genes related to fatty acid oxidation (*PPARα, ACOX1,* and *FGF21*). In addition, similar results were observed for SREBP1, PPARα, and FGF21 protein levels. The effects of (+)-dehydrovomifoliol were partially reversed by treatment with the PPARα antagonist GW6471, indicating the important role of the PPARα–FGF21 axis in the effects of (+)-dehydrovomifoliol. Based on its effects on hepatic lipogenesis and fatty acid oxidation signaling via the PPARα–FGF21 axis, (+)-dehydrovomifoliol isolated from *A. frigida* could be a useful early lead compound for developing new drugs for NAFLD prevention.

## Introduction

Non-alcoholic fatty liver disease (NAFLD) is a common chronic liver disease that is prevalent in China, affecting approximately 29% of the population when compared with the global prevalence of approximately 25%) (Loomba et al., 2021). The prevalence of NAFLD is clearly related to the growing epidemic of metabolic diseases, such as obesity and cardiovascular diseases (Bellentani, 2017). A subset (15–20%) of patients with NAFLD progresses to develop the more severe disease nonalcoholic steatohepatitis (NASH) (Tilg and Moschen, 2010). The pathogenesis of NAFLD involves a “two-hit” hypothesis; hepatic triglyceride (TG) accumulation is a key step of the first hit leading to NASH, which is always associated with hepatic steatosis, including lipogenesis, lipolysis, and fatty acid oxidation (Basaranoglu et al., 2013). Therefore, targeting lipid dysregulation is extremely important for developing additional complex treatment options for patients with NAFLD.

PPARα is a master regulator of lipid metabolism and ketogenesis in the liver (Rakhshandehroo et al., 2009). Fibroblast growth factor 21 (*FGF21*) is a direct target gene of PPARα and a key mediator of hepatic lipid metabolism (Badman et al., 2007). Because of its ability to not only directly modulate lipid metabolism in the liver but also to improve whole-body metabolic homeostasis, the PPARα–FGF21 axis has emerged as a promising target for the treatment of NAFLD and NASH. FGF21 reduces the expression of lipogenic genes, including *SCD1*, *FASN*, and *SREBP1*. *SREBP1* is a master regulator of the lipogenic transcriptional network that is overexpressed upon ingestion of a high-fat diet. (Gälman et al., 2008; Tillman and Rolph, 2020). Other studies have shown that activation of the PPARα–FGF21 pathway has protective effects against lipid accumulation and NAFLD and NASH development (Marino et al., 2016; Zeng et al., 2017). Although there are several pharmacological treatments for NAFLD, such as lipid-lowering agents, insulin sensitizers, and cytoprotective and antioxidant agents, their prolonged use is associated with a risk of adverse effects (Singh et al., 2015). Thus, innocuous, more effective therapeutic agents—possibly of natural origin—are needed to treat hepatic TG accumulation in patients with NAFLD.

*Artemisia frigida*, a perennial herb of the family Compositae, is widely used for the treatment of numerous human diseases, including jaundice and cold, in China. As a traditional medicine, it has a long history of use for the treatment of arthrosis, carbuncles, and bleeding (Jiggemudedan, 1999). Modern pharmacological studies have shown that it has multiple bioactivities, including anticancer, anti-inflammatory, and antioxidant effects (Qinghu Wang et al., 2015; Olennikov et al., 2019). Phytochemistry research showed that *A. frigida* mainly contains terpenes and flavonoids. As part of our ongoing exploration aimed at discovering bioactive natural products for treating NAFLD from *artemisia* plants, the dried aerial parts of *A. frigida* were extracted and fractionated to yield (+)-dehydrovomifoliol (**1**) and eupatilin (**2**).

In summary, the present study described (+)-dehydrovomifoliol, isolated from a traditional ethnic medicine, alleviates oleic acid-induced lipid accumulation in HepG2 cells *via* the PPARα–FGF21 pathway. These results are available on its mechanism of action underlying this activity, and are very helpful to provide clues and useful exploration for drug research based on natural products especially terpenoids.

## Materials and Methods

### Plant Material

The aerial parts of *A. frigida* were originally collected from Sichuan province, China in July 2017 and identified by Dr. Xudong Zhou, who is one of the corresponding authors. A voucher specimen (No. LH201707) was deposited in the Herbarium of Innovative Materia Medica Research Institute, Hunan University of Chinese Medicine.

### Extraction and Isolation

Air-dried and powdered aerial parts of *A. frigida* (2.2 kg) were immersed in 95% aqueous EtOH, four times for 3 h, each. The EtOH extract was concentrated; suspended in water; and sequentially partitioned with petroleum ether (51.2 g), methylene dichloride (26.9 g), ethyl acetate (7.43 g), and *n*-BuOH (34.6 g). The methylene dichloride (CH_2_Cl_2_) fraction was separated by chromatography using a silica gel column (200–300 mesh; petroleum/EtOAc, 1:0→0:1) to yield seven fractions (Fr. A–G) and then Fr. E was separated using a MCI column (MeOH/H_2_O, 40:60→90:20) to yield seven major sub-fractions (Fr. E1–E7). Fr. E7 was re-separated by semi-preparative HPLC (MeOH/H_2_O 41:59, 2.0 ml/min) to yield compounds **1** (5.2 mg, retention time (Rt): 26.0 min) and **2** (6.7 mg, Rt: 38.1 min). Their structures were determined by comparing with data in the available literature (González, et al., 1988; Kisiel et al., 2003; Kim et al., 2004; Suleimenov et al., 2005), including NMR spectra and optical rotation values. (+)-Dehydrovomifoliol **1**) was isolated from this plant for the first time.

### Cell Culture

HepG2 cells were cultured in a 5% CO_2_ incubator under a humidified atmosphere at 37°C for 2–3 days. The cells were grown in Advanced Roswell Park Memorial Institute (RPMI) 1,640 medium (Thermo Fisher Scientific, Waltham, MA, United States) supplemented with 10% fetal bovine serum (ThermoFisher) and 1% penicillin-streptomycin (Solarbio, Beijing, China). Upon reaching a cell density of 70–80%, the cells were subcultured at a ratio of 1:4.

### Cell Viability Assay

For the toxicity screening, HepG2 cells were seeded in 96-well plates at a density of 1 × 10^4^ cells/well. WST-8 reagent (10 μl; Beyotime, Shanghai, China) was added to each well and incubated at 37°C for 2 h. Finally, the absorbance at 450 nm was measured using a microplate reader. The effects on cell growth are reported as a percentage of the control.

### Oil Red O Staining

Oil red O (ORO) was purchased from Sigma (St. Louis, MO, United States). In brief, HepG2 cells were fixed with 4% paraformaldehyde for 30 min and then stained with 0.5% ORO (w:v) for 15 min at 20 °C–25°C. Nuclei were stained with hematoxylin for 10 min. All stained sections were examined via light microscopy.

### Measurement of Intracellular TGs

To measure the intracellular TG content in HepG2 cells, the cells were lysed with 0.1% Triton X-100 (Solarbio). The resulting cell lysates were used to assess TG content using a commercial kit (Jiancheng, Nanjing, China) according to the manufacturer’s instructions.

### RT-qPCR Analysis

Total RNA was isolated from the HepG2 cells with different treatment using the RNeasy mini kit (Qiagen, Hilden, Germany) according to the manufacturer’s instructions. Then, the iScript cDNA Synthesis Kit (Bio-Rad, Hercules, CA, United States) was used to reverse transcribe the extracted RNA. The resulting cDNA was used for real-time qPCR amplification with specific primers and iTaq universal SYBR Green Supermix (Bio-Rad) according to the manufacturer’s instructions. The reaction was performed in triplicate using the StepOnePlus Real-Time PCR system (Thermo Fisher Scientific), and the resulting values were normalized to those of a housekeeping gene (β-actin or glyceraldehyde 3-phosphate dehydrogenase [GAPDH]) (Chen et al., 2019).

### Western Blotting Analysis

HepG2 cells were seeded at a density of 1 × 10^5^ cell/ml in 25 cm^2^ flasks and before induction with oleic acid (OA) for 24 h, HepG2 cells were pretreated with/without 10 μM GW6471 (HY-15372, MCE, Shanghai, China) for 2 h and with different concentration of compound **1** for 30 min. Then, the cells were harvested and lysed with RIPA lysis buffer (Thermo Fisher Scientific) containing 1× protease inhibitor and phosphatase inhibitor cocktails (Roche Molecular Biochemicals, Basel, Switzerland). The Bio-Rad protein assay (Bio-Rad) was used to measure protein content, and equal amounts of protein were separated by sodium dodecyl sulfate (SDS)-PAGE (NuPage, Bis-Tris Gel 4–12%; Thermo Fisher Scientific). The proteins were transferred onto polyvinylidene difluoride membranes and then immunoblotted with specific primary antibodies against SREBP1 (# ab28481, 1:2000), PPARα (# ab171941, 1:2000), FGF21 (# ab126285, 1:2000), and GAPDH (# AP0063, 1:5,000) at 4°C overnight with gentle shaking. Bound antibodies were detected using the Immobilon Western Chemiluminescent kit (Millipore, Billerica, MA, United States). Images were taken with an ImageQuant LAS 4000 mini (Fujifilm, Tokyo, Japan) (Chen et al., 2019).

### Statistical Analysis

Statistical analyses were performed using GraphPad Prism 5 (GraphPad Software, Inc, San Diego, CA, United States). Values shown are the means ± Standard Error of Mean (SEM). The data were analyzed using one-way Student’s *t*-test, and *p* values less than 0.05 (^*^ *p* < 0.05, ^**^ *p* < 0.01, ^***^ *p* < 0.001) were considered statistically significant (Lee et al., 2019).

## Results

### Isolation and Structure Elucidation

Through bioassay-guided fractionation of the 95% aqueous EtOH extract of *A. frigida* aerial parts, we found that the methylene dichloride (CH_2_Cl_2_) fraction exerted lipid accumulation decreasing effects in OA-induced HepG2 cells (data not shown). Further separation of the CH_2_Cl_2_ fraction via activity-guided chromatographic methods yielded (+)-dehydrovomifoliol (**1**), which was isolated from this plant for the first time, and a known compound named eupatilin (**2**).

### (+)-Dehydrovomifoliol Alleviates OA-Induced Excessive Lipid Accumulation in HepG2 Cells

**FIGURE 1:**
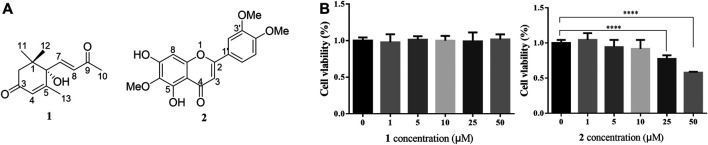
Chemical structures of **1** and **2**, and their cytotoxicity analysis. **(A)** Chemical structures of compounds **1** and **2** isolated from the methylene dichloride (CH_2_Cl_2_) fraction of *artemisia frigida* aerial parts. **(B)** Changes in the viability of HepG2 cells treated with different concentrations of compound **1** or **2**. Cell viability as assessed using the CCK-8 assay at 24 h post-treatment. Data shown in the bar chart are expressed as the mean ± SEM (*n* = 3). ****p < 0.0001.

**FIGURE 2:**
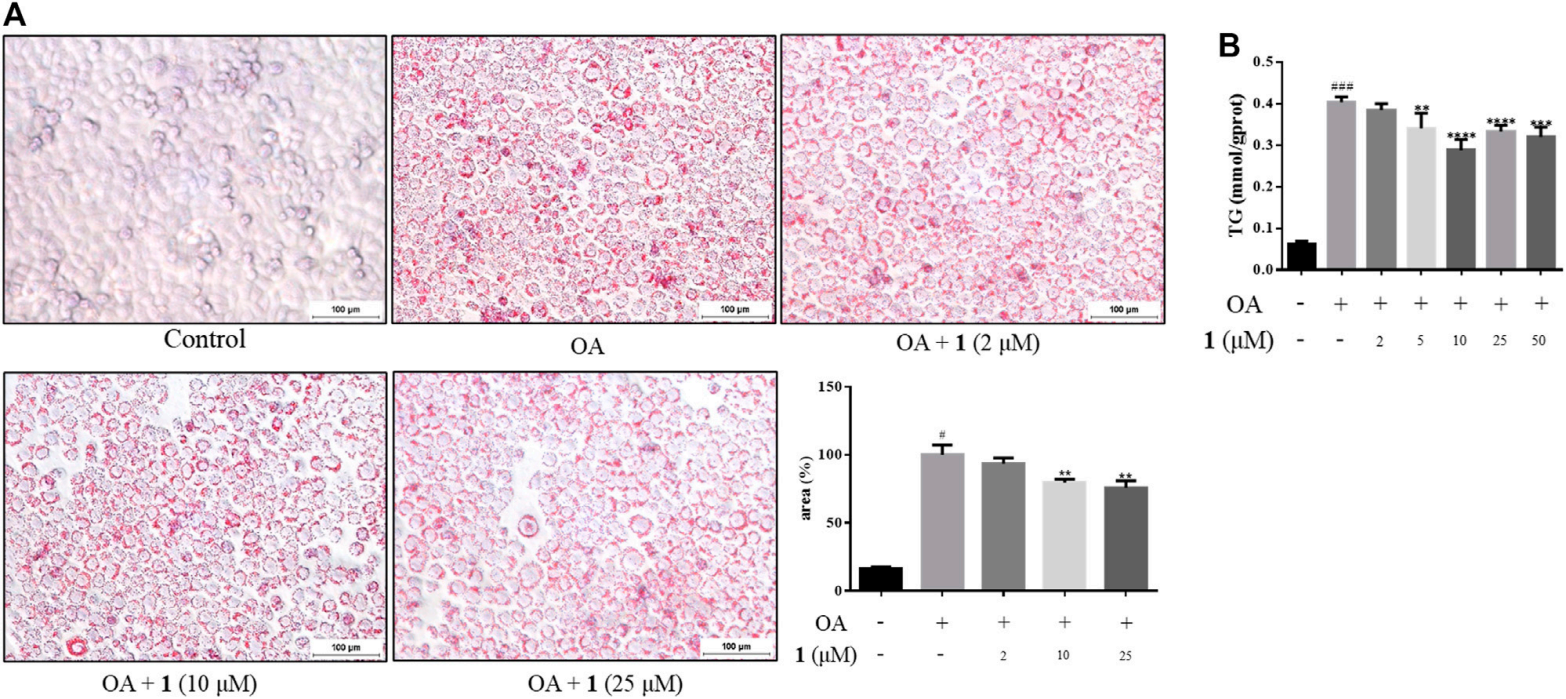
(+)-Dehydrovomifoliol alleviates OA-induced excessive lipid accumulation in HepG2 cells. HepG2 cells were pretreated for 30 min with different concentrations of compound **1** and then stimulated with 10 μM oleic acid (OA). Then, at 24 h post-treatment **(A)** Oil red O staining images (100×) and the percentages of HepG2 cells containing lipid droplets. **(B)** TG contents were analyzed using the TG detection assay. Data shown in the bar chart are expressed as the mean ± SEM (*n* = 3). ^#^ *p* < 0.05, ^# # #^ *p* < 0.001 vs the control group; ^*^ *p* < 0.05, ^**^ *p* < 0.01, ^***^ *p* < 0.001, ^****^ *p* < 0.0001 vs the OA-induced group.

As shown in Figure 1B, the CCK-8 assays demonstrated that compound **1** was not toxic to HepG2 cells at concentrations lower than 50 μM whereas compound **2** was cytotoxic at 25 and 50 μM. Since the cytotoxic effects of **2,** we chose **1** for further study. The results of the TG assay and oil red staining revealed that OA increased the lipid contents in HepG2 cells. However, pretreatment with (+)-dehydrovomifoliol significantly reduced OA-induced lipid accumulation (Figures 2A,B), and the effect at 10 μM was not significantly different than that at 25 or 50 μM. Therefore, 10 μM was regarded as a safe concentration and was chosen for subsequent experiments. Oil red staining also showed a significant decrease in lipid droplets after treatment with 10 μM (+)-dehydrovomifoliol. Importantly, these beneficial effects in OA-induced HepG2 cells were dependent on (+)-dehydrovomifoliol dose.

### Changes in Gene and Protein Expression Levels in OA-Induced HepG2 Cells After (+)-Dehydrovomifoliol Treatment

**FIGURE 3:**
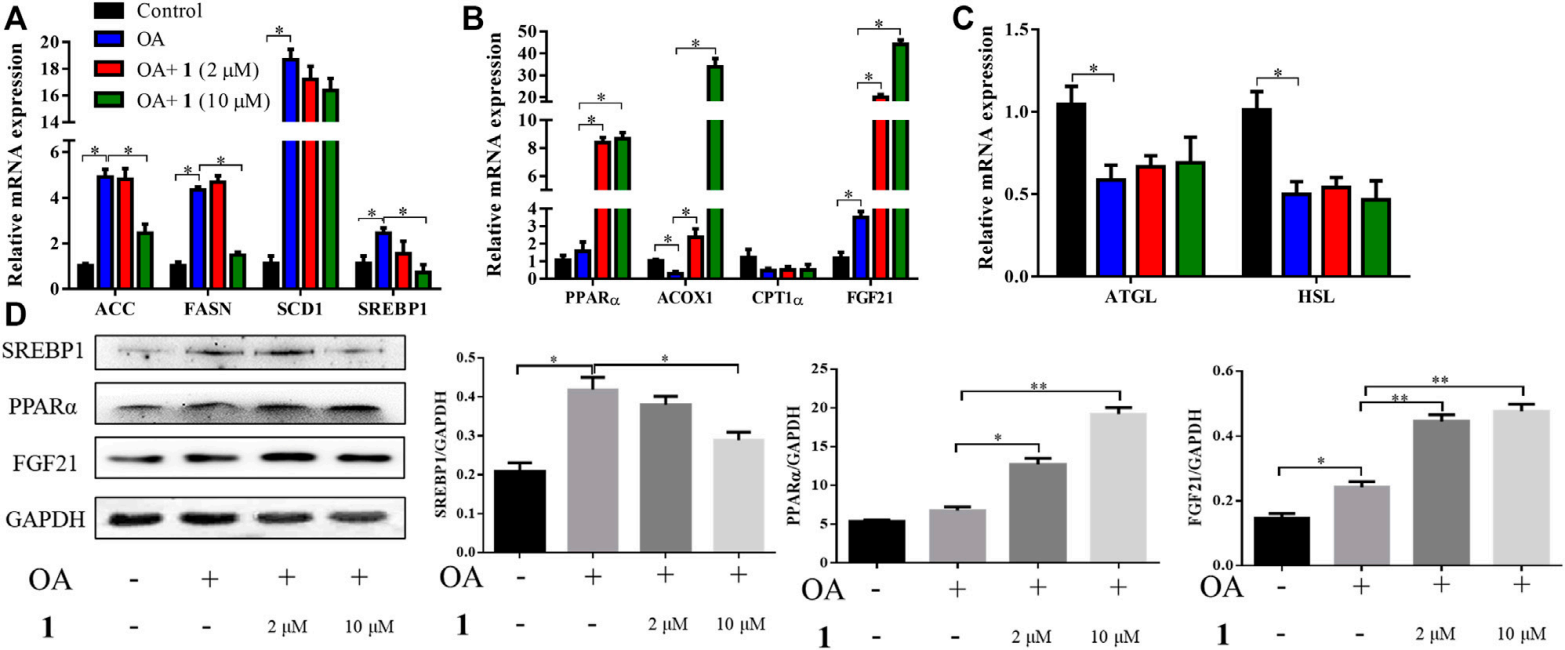
(+)-Dehydrovomifoliol modulates lipid metabolism genes in HepG2 cells. HepG2 cells were pretreated for 30 min with different concentrations of **1** and then stimulated with 10 μM oleic acid (OA) for 24 h. Then, the mRNA levels of genes related to lipid metabolism, including **(A)** lipogenesis **(B)** fatty acid oxidation, and **(C)** lipolysis, were detected using RT-QPCR. **(D)** Western blotting analysis of Srebp1, PPARα, and FGF21 protein levels in HepG2 cells and quantification by densitometric scanning. Data shown in the bar chart are expressed as the mean ± SEM (*n* = 3). ^*^ *p* < 0.05, ^**^ *p* < 0.01.

Excessive lipid accumulation is caused by either overactivation of lipogenesis or impairment of fatty acid oxidation and lipolysis. Thus, to elucidate the molecular events underlying the beneficial effects of (+)-dehydrovomifoliol, RT-qPCR analysis was used to quantify the mRNA expression levels of hepatic genes involved in these processes. As shown in Figures 3A–C, significant increases in the mRNA expression levels of lipogenesis-related genes that promote the synthesis of *de novo* monounsaturated fatty acids, such as *ACC*, *FASN*, *SCD1*, and *SREBP1*, occurred after OA stimulation whereas significant decreases in *SREBP1*, *ACC*, and *FASN* mRNA expression occurred after pretreatment with (+)-dehydrovomifoliol. Furthermore, the mRNA expression levels of three genes that exert lipid-lowering effects—*PPARα*, *ACOX1*, and *FGF21*—were significantly increased after pretreatment with (+)-dehydrovomifoliol. Administration of OA caused a drastic reduction in the mRNA expression levels of lipolysis-associated genes. However, these effects on mRNA expression levels were not observed after pretreatment with (+)-dehydrovomifoliol. Similar results were observed with SREBP1, PPARα, and FGF21 protein expression levels using western blotting (Figure 3D). These results indicate the PPARα–FGF21 axis is the potential target of (+)-dehydrovomifoliol.

### Treating Cells With a PPARα Antagonist Attenuates the Effects of (+)-Dehydrovomifoliol on OA-Induced Lipid Accumulation in HepG2 Cells

**FIGURE 4:**
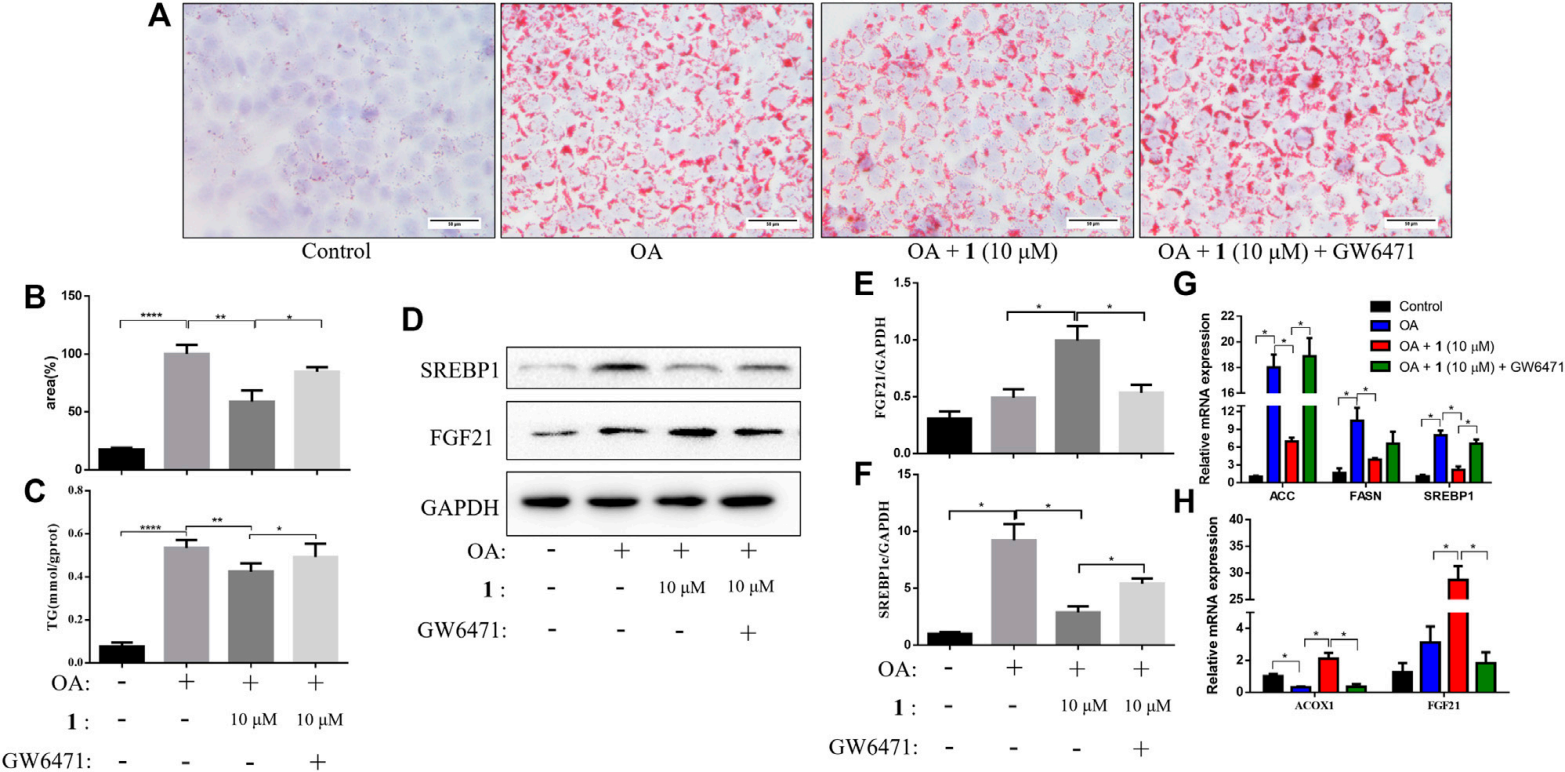
PPARα antagonist attenuates the effects of (+)-dehydrovomifoliol on OA-induced lipid accumulation in HepG2 cells. Before induction with oleic acid (OA) for 24 h, HepG2 cells were pretreated with 10 μM GW6471 for 2 h and with 10 μM compound **1** for 30 min. **(A)** Oil red O staining images (200×) and **(B)** the percentages of HepG2 cells containing lipid droplets. (**C**) Triglyceride (TG) levels were analyzed using the TG detection assay. **(D**, **E**, **F)** Western blotting analysis of SREBP1 and FGF21 protein levels in HepG2 cells and quantification by densitometric scanning. **(G)**, **(H)** mRNA levels of *ACC*, *FASN*, *SREBP1*, *ACOX1*, and *FGF21* were detected using RT-QPCR. Data shown in the bar chart are expressed as the mean ± SEM (*n* = 3). ^*^ *p* < 0.05, ^**^ *p* < 0.01, ^****^ *p* < 0.0001.

PPARα is an important nuclear factor that regulates numerous hepatic lipid metabolism genes including *FGF21*, which is one of the most important effectors (Inagaki et al., 2007; Vernia et al., 2014; Huang et al., 2017). We examined TG content and lipid accumulation in OA-induced HepG2 cells pretreated with GW6471 (a PPARα inhibitor) before (+)-dehydrovomifoliol treatment to further examine the role of the PPARα–FGF21 axis in (+)-dehydrovomifoliol-mediated protection against lipid accumulation. As shown in Figure 4, administration of GW6471 reversed the decrease in lipid accumulation caused by (+)-dehydrovomifoliol. In addition, the improvements in the mRNA expression levels of *SREBP1*, *ACC*, *FASN*, *ACOX1*, and *FGF21* were significantly reversed by GW6471 administration. Similar results were observed for the levels of SREBP1C and FGF21 proteins. These findings suggest that suppression of the PPARα–FGF21 axis partially abrogated the restoration effects of (+)-dehydrovomifoliol on lipid metabolism and the mRNA expression levels of related genes.

**FIGURE 5:**
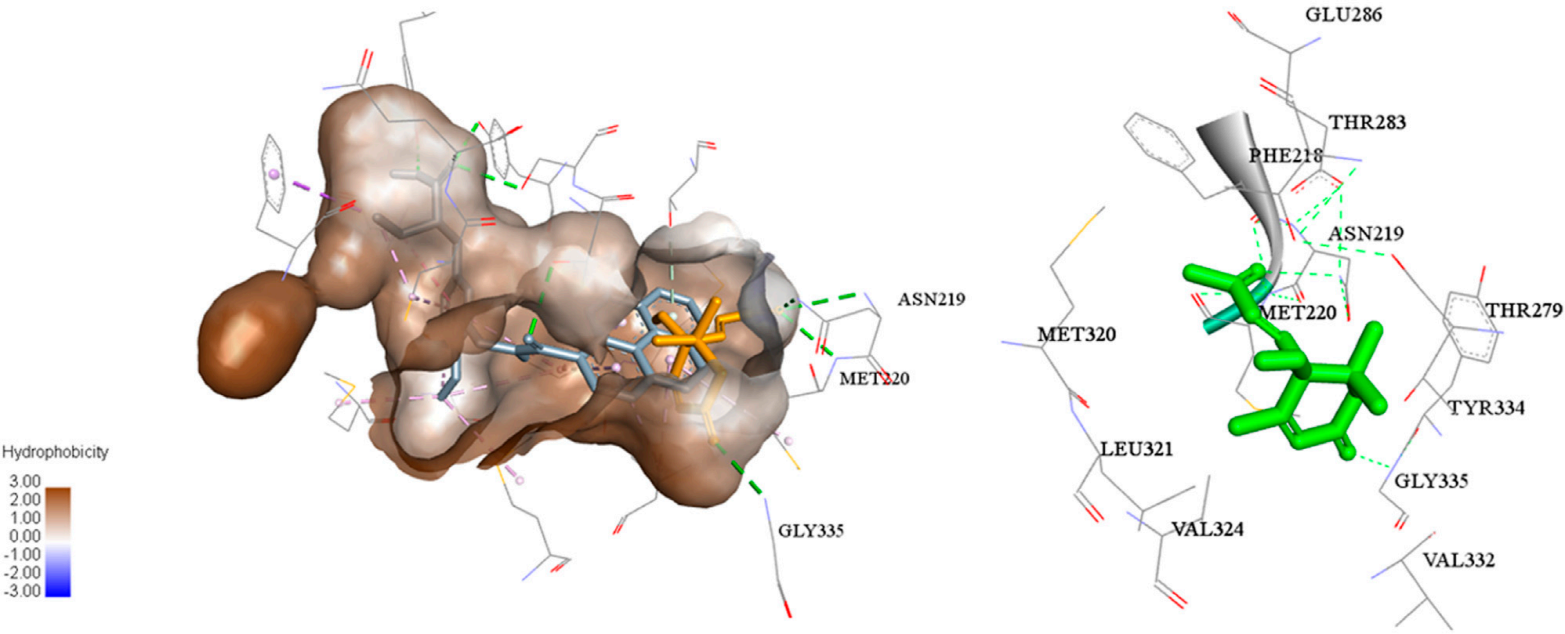
Molecular docking model of compound **1** binding to PPARα.

**FIGURE 6:**
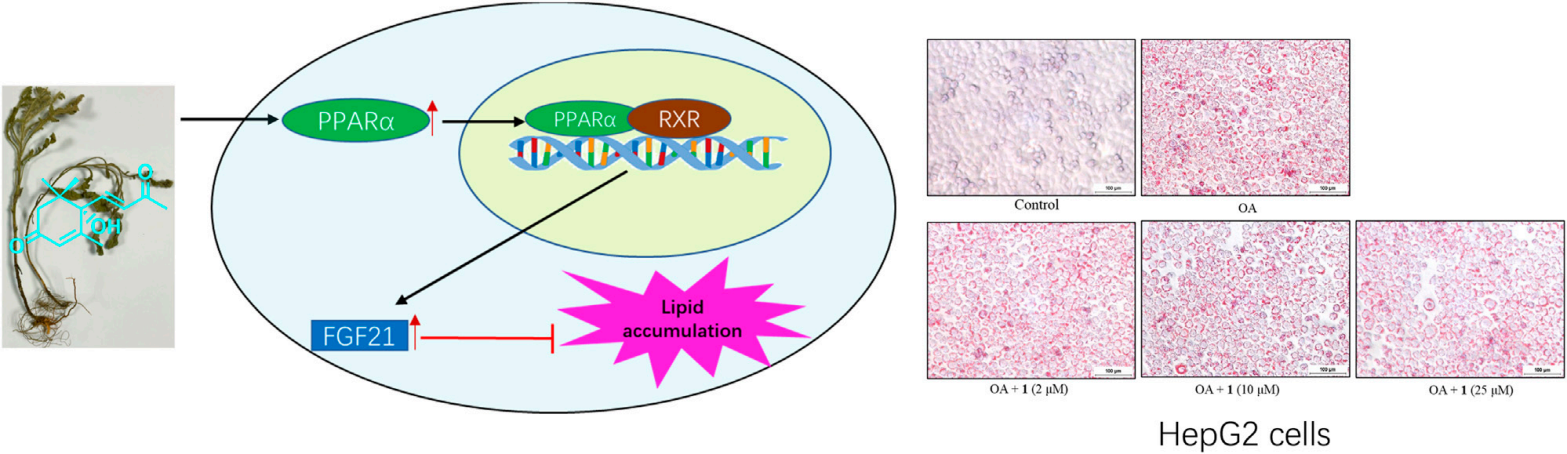
(+)-Dehydrovomifoliol activated the PPARα-FGF21 pathway to alleviate lipid accumulation.

In addition, a molecular docking study (Figure 5) showed that the C-3 and C-9 carbonyl in (+)-dehydrovomifoliol formed hydrogen bond donors and/or acceptors with some amino acid residues of PPARα (Gly335, Met220, and Asn219) and thus increased the activity of the compound (Kuwabara et al., 2012). However , the space around these oxygen atoms in PPARα was large enough for further optimization to improve the biological activity of (+)-dehydrovomifoliol, suggesting its potential as a lead compound (Figure 6).

## Discussion

Hepatic lipid accumulation is central to the pathogenesis of NAFLD and NASH, and it is also a risk factor for many other chronic metabolic perturbations. Natural products have diverse biological activities for diseases associated with lipid accumulation (Vermaak et al., 2011). The previous research found saponins from *Momordica charantia* could reduce fat accumulation by targeting multiple lipid mechanisms containing the *sbp-1*/*mdt-15* pathway (Liu et al., 2019). Auraptene, a natural compound, showed the inhibition of P-glycoprotein expression, which widely existed in liver (Nabekura et al., 2020). Ganoderic acid A, a triterpene from *Ganoderma lucidum*, ameliorated lipid metabolism in hyperlipidemic mice fed a high-fat diet, and could regulated the mRNA levels of hepatic genes involved in fatty acid metabolism (Guo et al., 2020). Another natural terpenoid ursolic acid reduced ROS accumulation in a nematode model (Naβ et al., 2021). These studies exhibited terpenoids had potential anti-fat accumulation activity.

Using an *in vitro* model of lipid accumulation (OA-induced HepG2 cells), we showed that (+)-dehydrovomifoliol alleviated intracellular lipid accumulation. Mechanistically, the results of our study indicate that (+)-dehydrovomifoliol significantly alters multiple pathways involved in lipid homeostasis. More specifically (+)-dehydrovomifoliol suppresses the expression of genes modulating lipogenesis and TG synthesis and storage and enhances the transcription of genes involved in fatty acid oxidation and TG secretion, suggesting that (+)-dehydrovomifoliol has broad functions in lipid metabolism homeostasis.

Lipid homeostasis is dependent on the balance between lipogenesis, lipolysis, and fatty acid oxidation (Lelliott and Vidal-Puig, 2004). Lipogenesis is controlled by a cluster of transcriptional factors, such as SREBP1, that drive the transcription of target genes including *FASN*, *ACC*, and *SCD1* (Linden et al., 2018). PPARα is a ligand-activated factor that plays an important role in regulating fatty acid and ketogenesis. Studies conducted in animals have consistently reported the importance of PPARα expression in steatosis and NASH. For example, PPARα null mice develop aggressive steatohepatitis when fed a high-fat diet or methionine and choline-deficient diet (Ip et al., 2003; Abdelmegeed et al., 2011). Activation of PPARα reverses high-fat diet-induced hepatocellular injury and liver inflammation and improves insulin sensitivity (Larter et al., 2012). FGF21, a direct target of PPARα, is a metabolic regulator that strongly affects both lipid and glucose homeostasis. These findings are also in agreement with studies showing that PPARα null mice are deficient in FGF21 and that treating these mice with FGF21 improves hypertriglyceridemia and hypoketonemia (Lundåsen et al., 2007). In this study, we found that OA increased the mRNA expression levels of *SREBP1*, *ACC*, and *FASN*; however (+)-dehydrovomifoliol treatment reversed these increases. Administration of (+)-dehydrovomifoliol significantly increased FGF21 mRNA and protein expression levels in OA-induced HepG2 cells and decreased lipid accumulation. In contrast, co-treatment with the PPARα antagonist GW6471 blocked these effects. This finding suggests the involvement of signaling via the PPARα–FGF21 axis in (+)-dehydrovomifoliol-mediated alleviation of OA-induced excessive lipid accumulation. More importantly, modulation of PPARα and FGF21 at both the mRNA and protein levels was dependent on (+)-dehydrovomifoliol dose. Thus, we concluded that PPARα might be a target of (+)-dehydrovomifoliol, which was confirmed by the antagonistic effect of GW6471 pretreatment. Finally, a molecular docking study showed that (+)-dehydrovomifoliol could be useful as an effective scaffold for a novel series of PPARα agonists. The mechanism underlying this activation remains a question for further investigations, including structural and physiological binding analyses. Drugs targeting PPARα, such as fibrates, have been developed and used in clinical applications for a long time (American Diabetes Association 2016). Although fibrates possess satisfactory properties for managing lipid homeostasis, most of them have adverse or side effects (Myerson, 2016; Julia et al., 2017). Therefore, more potent and possibly more selective agonists targeting PPARα are urgently needed for treating NAFLD and NASH (Pawlak et al., 2014).

In conclusion, this study demonstrates that (+)-dehydrovomifoliol protects hepatocytes against OA-induced lipid accumulation through activation of the PPARα–FGF21 pathway and hence encourages the future use of (+)-dehydrovomifoliol derivatives for the prevention and/or treatment of NAFLD and NASH and their complications. Further studies need to be conducted to evaluate the lipid-lowering effects and toxicity of (+)-dehydrovomifoliol *in vivo* based on the doses used in this study.

## Data Availability

The original contributions presented in the study are included in the article/Supplementary Material, further inquiries can be directed to the corresponding authors.
