# Supplementary material for: (+)-Dehydrovomifoliol Alleviates Oleic Acid-Induced Lipid Accumulation in HepG2 Cells via the PPARα–FGF21 Pathway
Source: Front Pharmacol. 2021 Nov 19;12:750147. doi: 10.3389/fphar.2021.750147 (PMC8640464; doi:10.3389/fphar.2021.750147)
Supplement: Supplementary file 1 [file Presentation1.PPTX]

## Slide 1
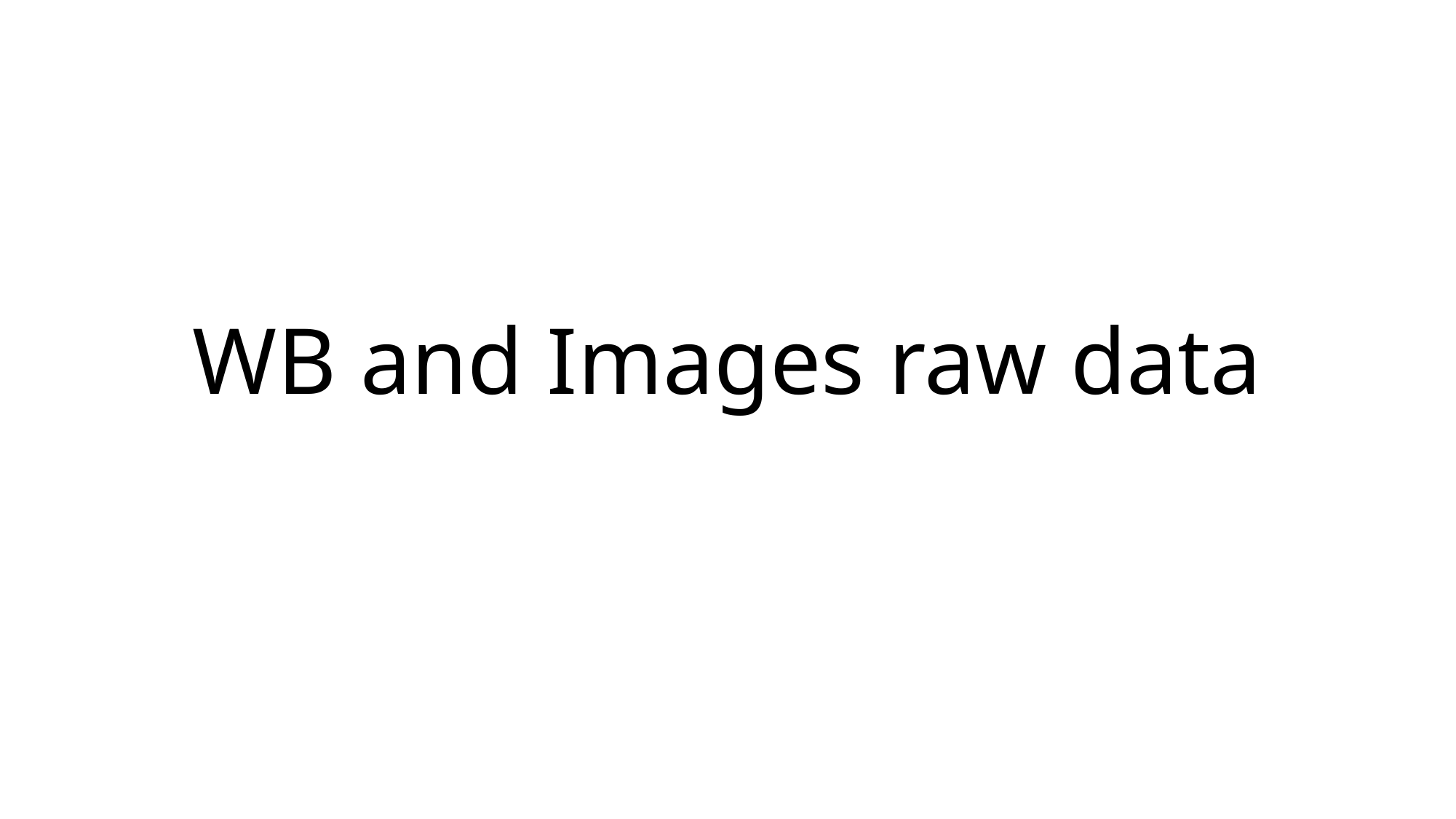

# WB and Images raw data

## Slide 2
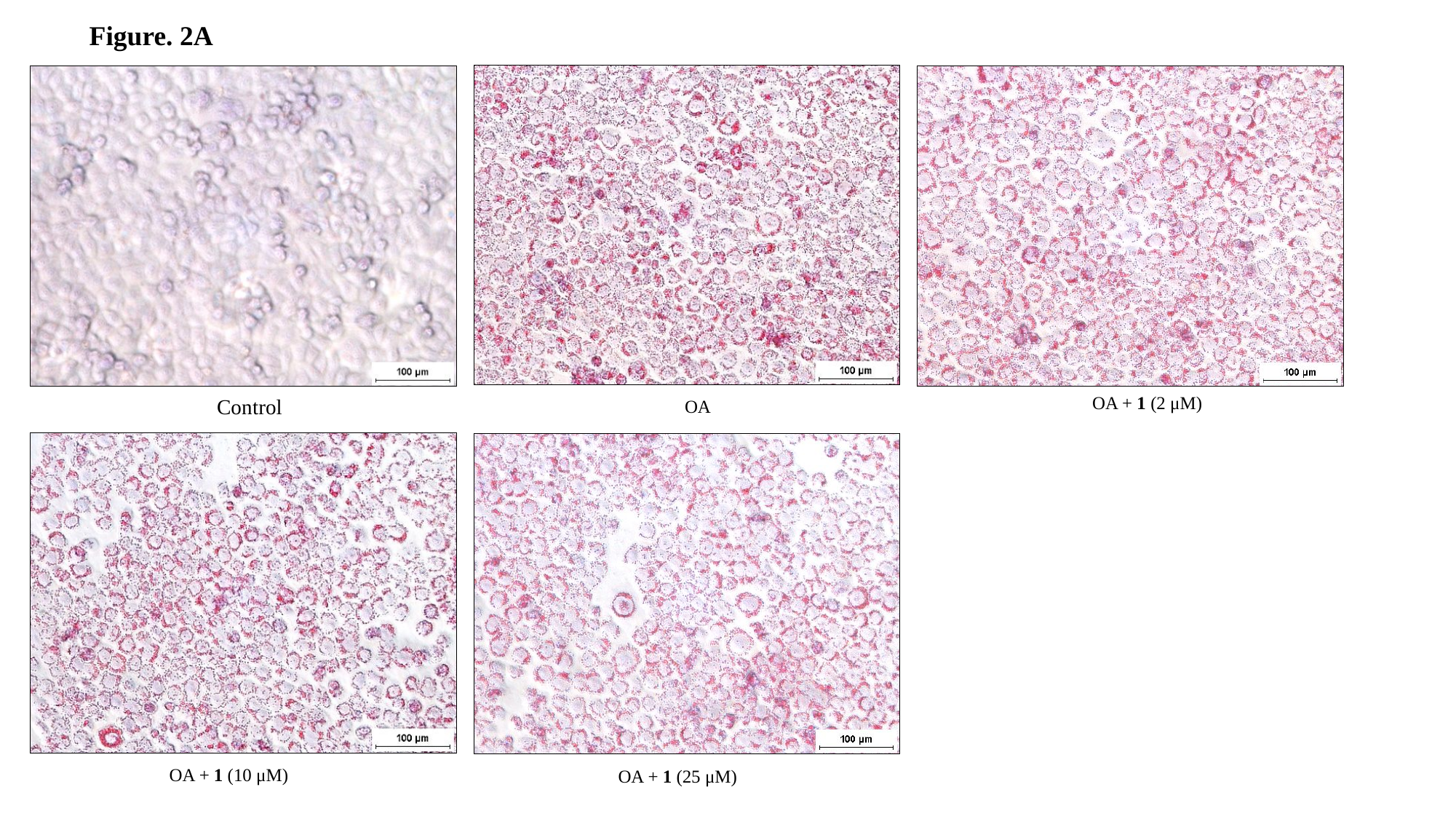

Figure. 2A
OA + 1 (2 μM)
Control
OA
OA + 1 (10 μM)
OA + 1 (25 μM)

## Slide 3
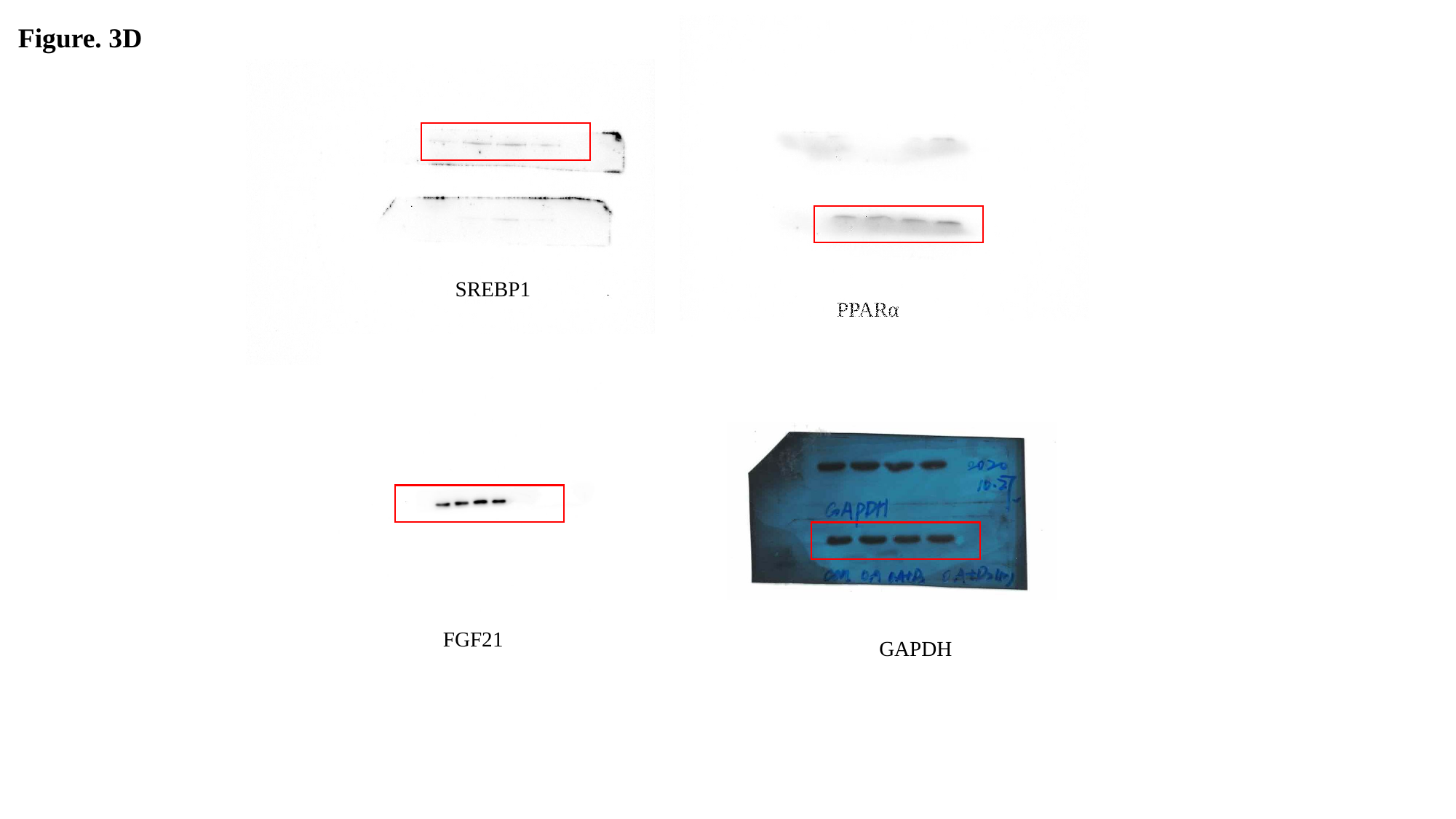

Figure. 3D
SREBP1
PPARα
FGF21
GAPDH

## Slide 4
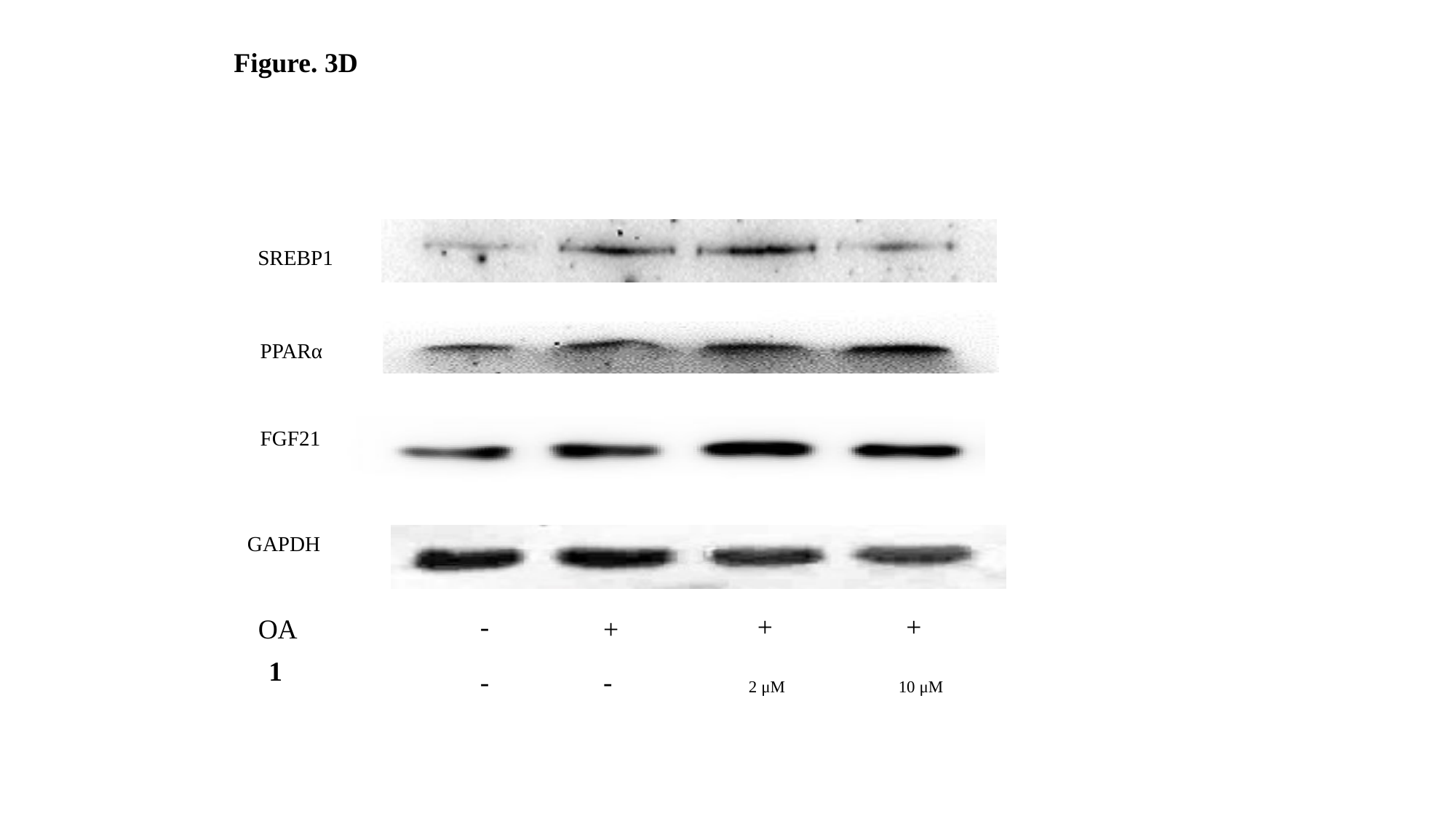

Figure. 3D
SREBP1
PPARα
FGF21
GAPDH
-
+
+
+
-
-
2 μM
10 μM
OA
1

## Slide 5
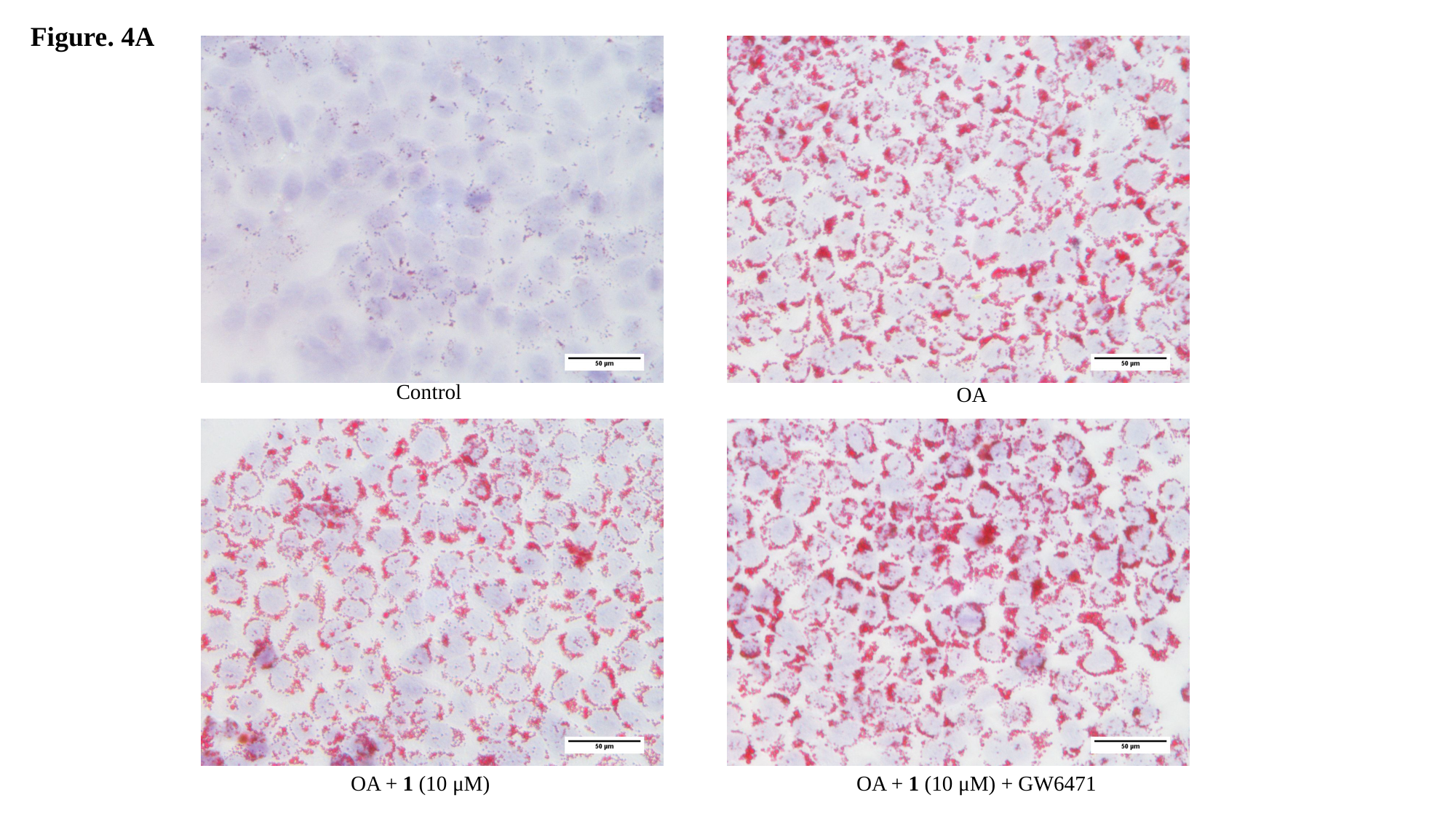

Figure. 4A
Control
OA
OA + 1 (10 μM)
OA + 1 (10 μM) + GW6471
