# Supplementary material for: (+)-Dehydrovomifoliol Alleviates Oleic Acid-Induced Lipid Accumulation in HepG2 Cells via the PPARα–FGF21 Pathway
Source: Front Pharmacol. 2021 Nov 19;12:750147. doi: 10.3389/fphar.2021.750147 (PMC8640464; doi:10.3389/fphar.2021.750147)
Supplement: Supplementary file 2 [file Presentation2.PPTX]

## Slide 1
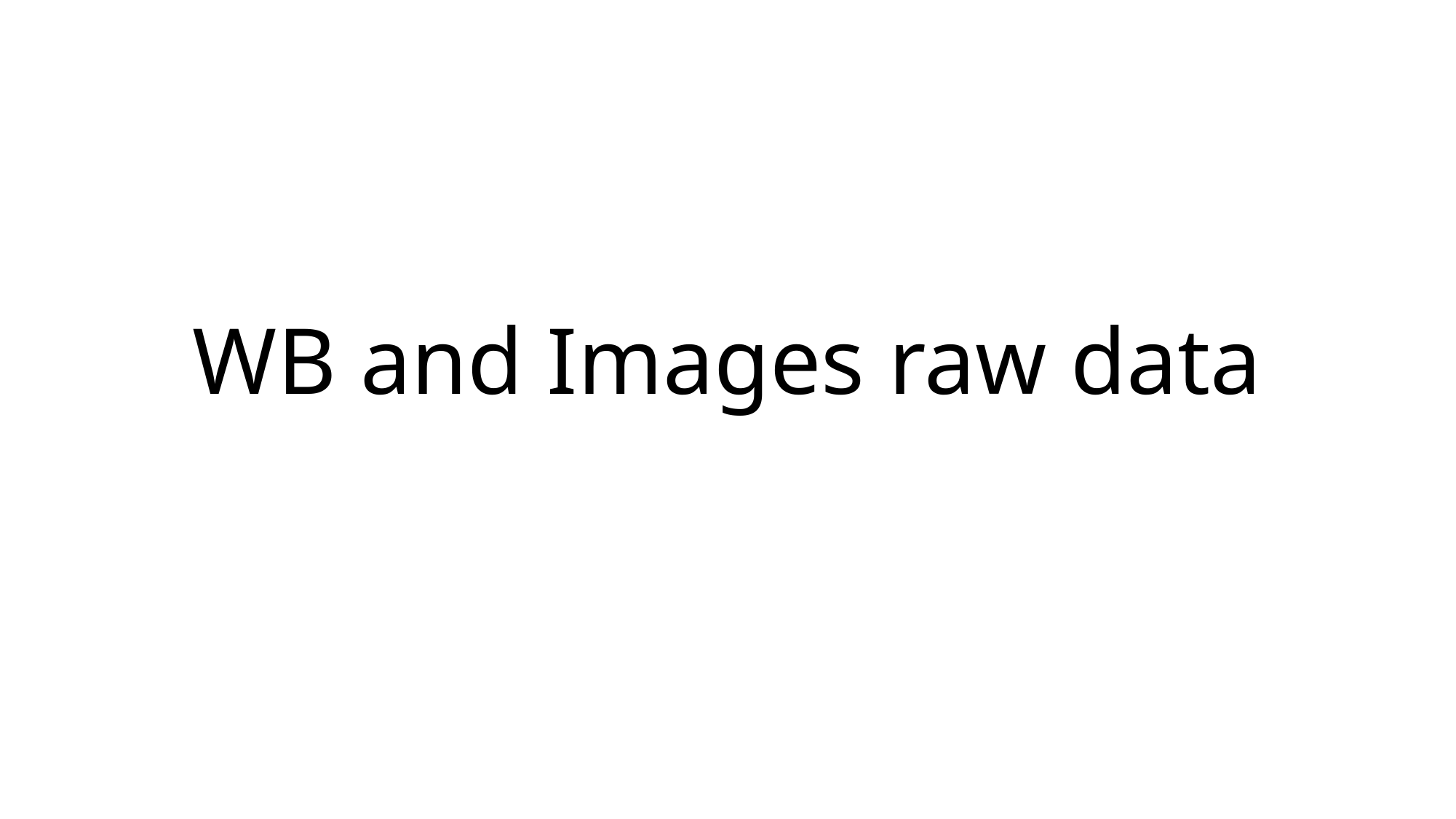

# WB and Images raw data

## Slide 2
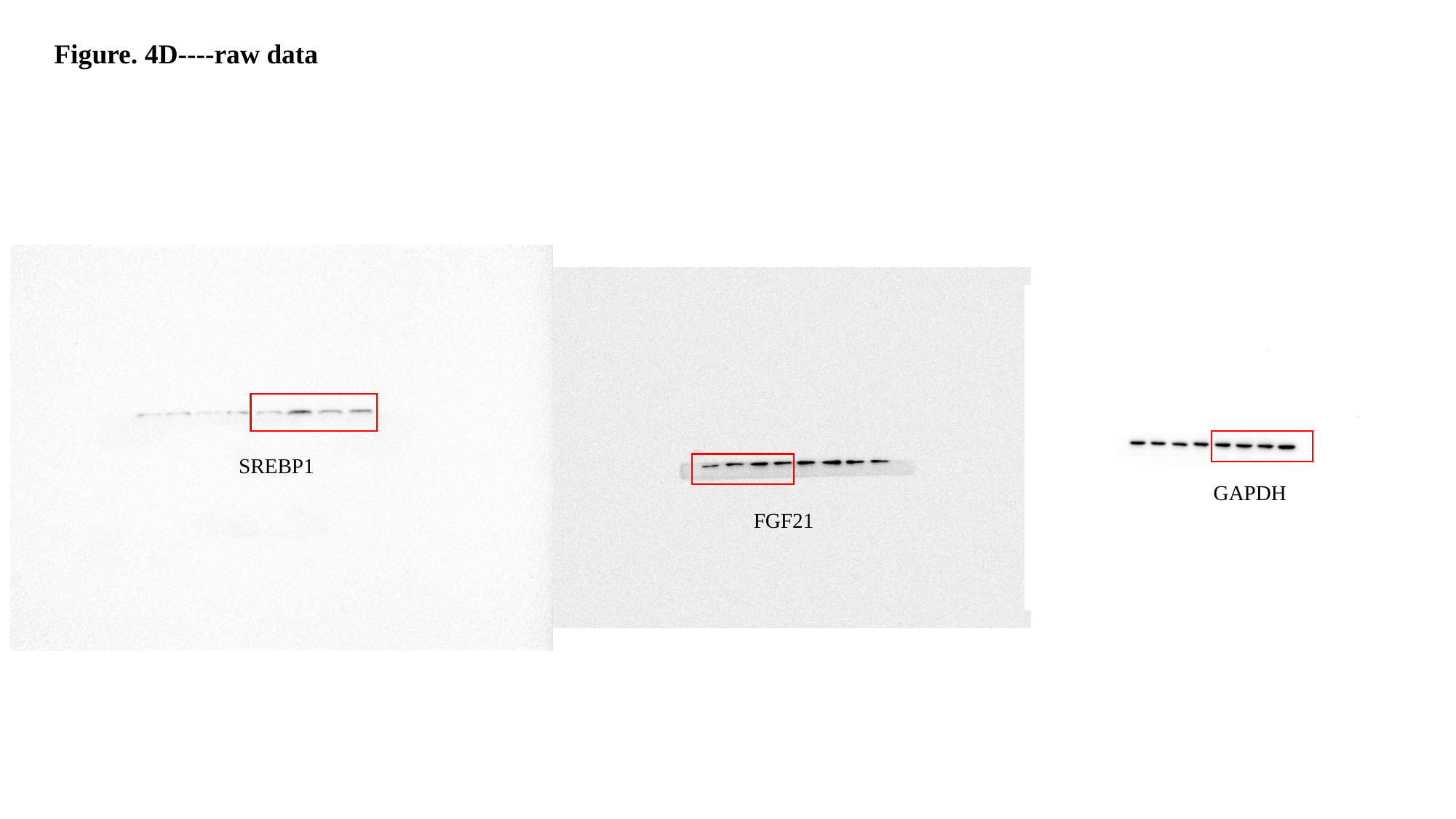

Figure. 4D----raw data
SREBP1
GAPDH
FGF21

## Slide 3
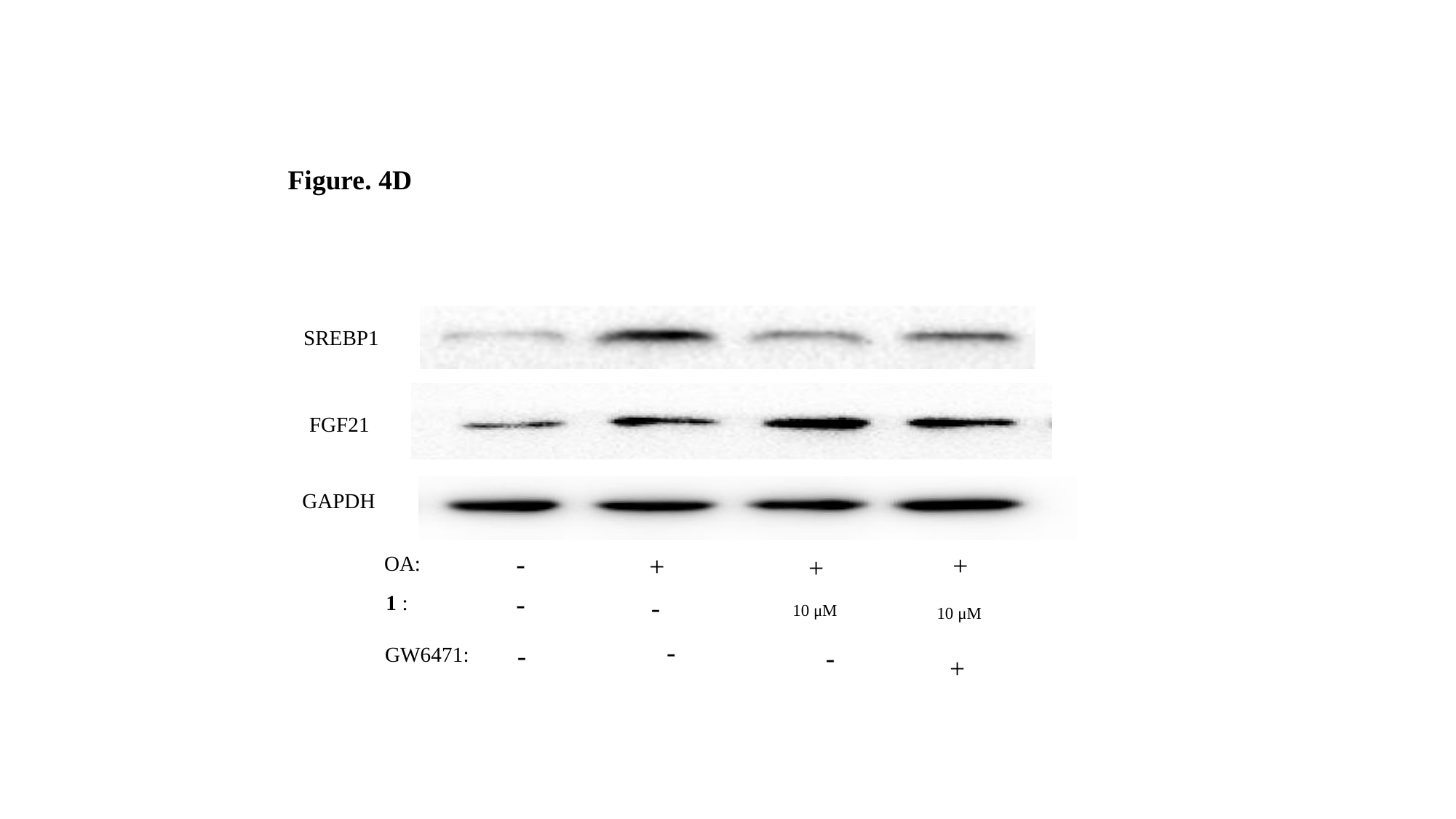

Figure. 4D
SREBP1
FGF21
GAPDH
-
+
+
OA:
+
-
1 :
-
10 μM
10 μM
-
-
-
GW6471:
+

## Slide 4
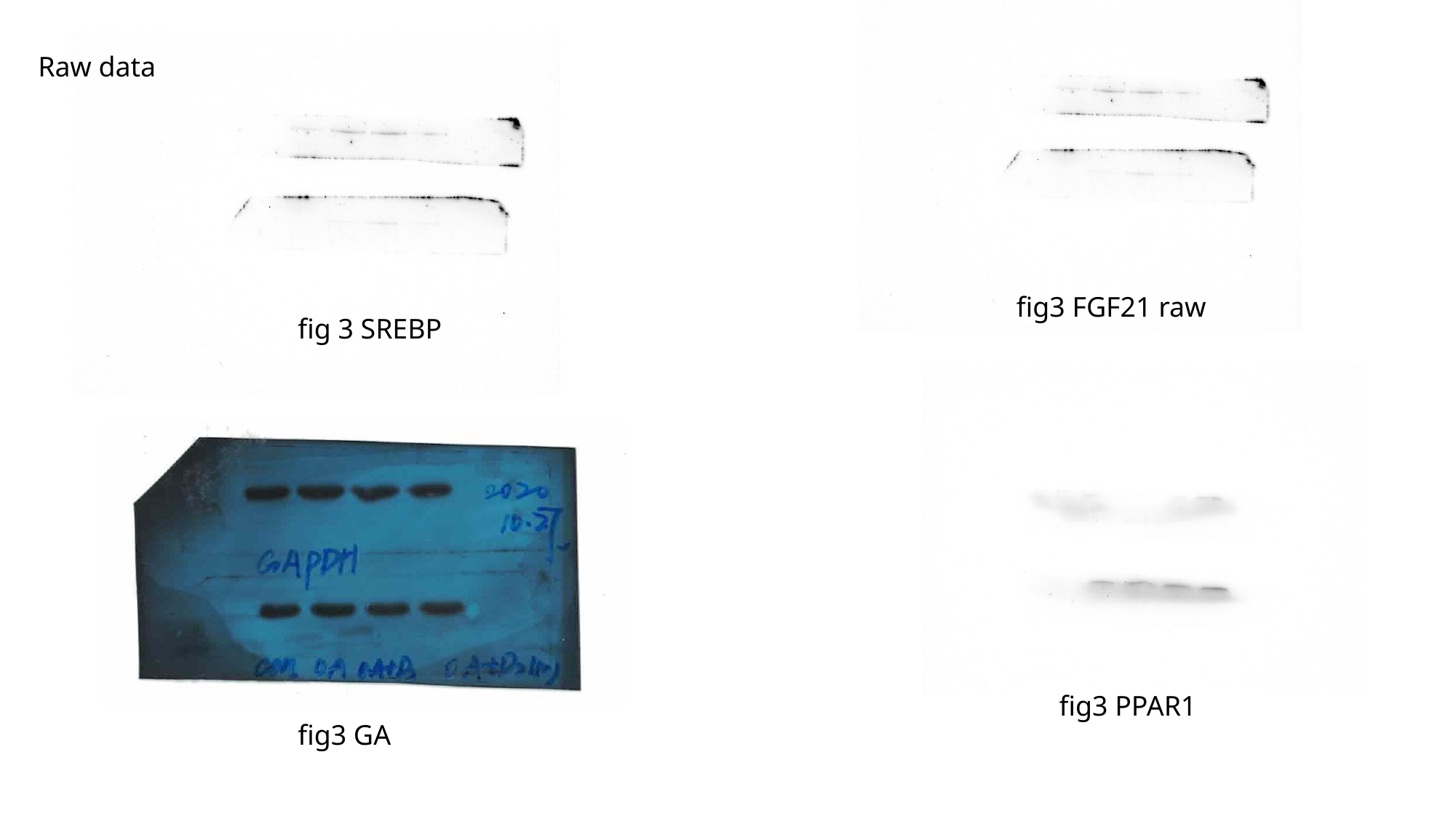

Raw data
fig3 FGF21 raw
fig 3 SREBP
fig3 PPAR1
fig3 GA

## Slide 5
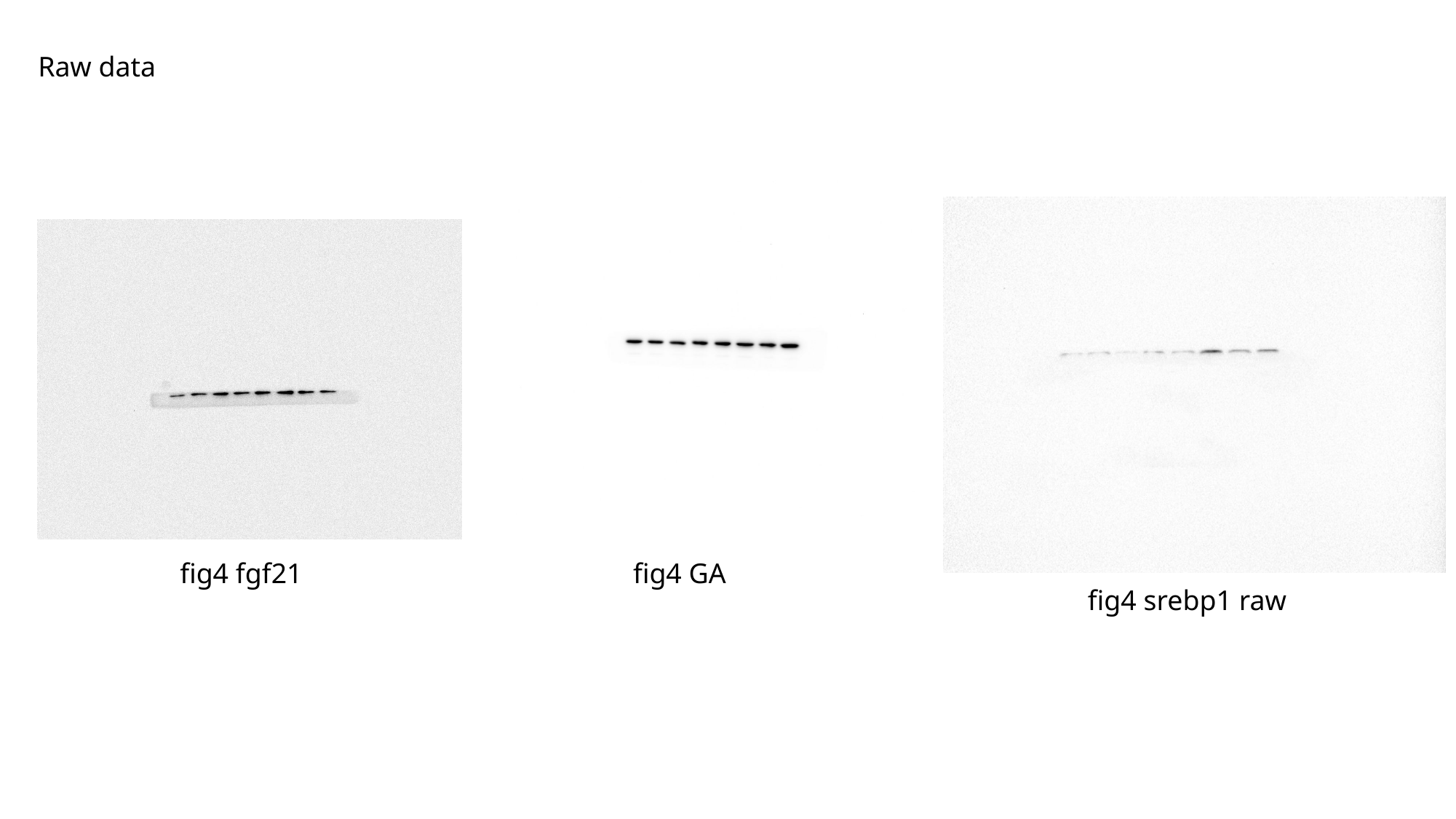

Raw data
fig4 fgf21
fig4 GA
fig4 srebp1 raw
