# Supplementary material for: (+)-Dehydrovomifoliol Alleviates Oleic Acid-Induced Lipid Accumulation in HepG2 Cells via the PPARα–FGF21 Pathway
Source: Front Pharmacol. 2021 Nov 19;12:750147. doi: 10.3389/fphar.2021.750147 (PMC8640464; doi:10.3389/fphar.2021.750147)
Supplement: Supplementary file 3 [file DataSheet1.docx]

**Supporting Information**

**(+)-Dehydrovomifoliol alleviates oleic acid-induced lipid accumulation in HepG2 cells via the PPARα–FGF21 pathway**

**Yiyuan Xi^1, 2^, Jujia Zheng^1^, Wei Xie^1^, Xiangwei Xu^4^, Namki Cho^2,*^, Xudong Zhou^3,*^, Xiaomin Yu^1,*^**

**1.Structure Characterization**

**(+)-Dehydrovomifoliol** (**1**): colorless oil; [α]_D_^20^ + 84.0 (*c* 0.03, MeOH); ^1^H-NMR (500 MHz, CDCl_3_) *δ*: 6.84 (1H, d, *J* = 15.7 Hz，H-7)，6.48 (1H, d, *J* = 15.7 Hz，H-8)，5.97 (1H, q, *J* = 1.3 Hz, H-4), 2.51 (1H, d, *J* = 17.2 Hz, H-2b), 2.35 (1H, d, *J* = 17.2 Hz, H-2a), 2.32 (1H, s, H-10), 1.89 (3H, d, *J* = 1.3 Hz, H-13), 1.12 (3H, s, H-11), 1.03 (3H, s, H-12). ^13^C-NMR (125 MHz, CDCl_3_) *δ*: 197.4 (C-3), 196.9 (C-9), 160.3 (C-5), 144.9 (C-7), 130.3 (C-8), 127.8(C-4), 79.3 (C-6), 49.6 (C-2), 41.4 (C-1), 28.4 (C-10), 24.3 (C-11), 22.9 (C-12), 18.7 (C-13). Based on a comparison of these NMR data and the optical rotation values with those in the literature (Kim, I., et al. 2004, Wanda Kisiel, et al. 2003), compound **1** was identified as (+)-dehydrovomifoliol.

**Eupatilin** (**2**): yellow powder; ^1^H-NMR (500 MHz, CDCl_3_) *δ*: 7.52 (1H, dd, *J* = 8.4, 2.0 Hz, H-6′), 7.34 (1H, d, *J* = 2.0 Hz, H-2′), 6.98 (1H, d, *J* = 8.4 Hz, H-5′), 6.61 (1H, s, H-8), 6.59 (1H, s, H-3), 4.05 (3H, s, OMe), 3.99 (3H, s, OMe), 3.97 (3H, s, OMe). ^13^C-NMR (125 MHz, CDCl_3_) *δ*: 182.8 (C-4), 164.1 (C-2), 155.0 (C-7), 153.1 (C-5), 152.3 (C-9), 152.1 (C-4′), 149.3 (C-3′), 130.3 (C-6), 123.7 (C-1′), 120.1 (C-6′), 111.1 (C-5′), 108.7 (C-2′), 105.7 (C-10), 104.0 (C-3), 93.4 (C-8), 60.9, 56.1, 56.1 (6,7,4′-OMe). The ^1^H and ^13^C NMR data were identical to those reported for eupatilin (AG González, et al. 1988, Suleimenov, E. M. et al. 2005).

**2.****Primer Sequences Used for Quantifying Gene Expression in HepG2 Cells**

**TABLE S1. Primer Sequences Used for Quantifying Gene Expression in HepG2 Cells**

| **Gene** | **Forward primer** | **Reverse primer** |
| --- | --- | --- |
| ACC | CAATATGGGGCAGTTAAAGCTT | CCTGAGTAATCTCACCCGATTT |
| FASN | GGAGGTGGTGATAGCCGGTAT | TGGGTAATCCATAGAGCCCAG |
| SCD1 | CGTCTGGAGGAACATCATTC | AGCGCTGGTCATGTAGTA |
| SREBP1 | GCTACCGGTCTTCTATCAATG | GCAAGAAGCGGATGTAGTC |
| PPARα | ATGCCAGTACTGCCGTTTTC | GGCCTTGACCTTGTTCATGT |
| ACOX1 | GCCTGCTGTGTGGGTATGTCATT | GTCATGGGCGGGTGCAT |
| CPT1α | AGATCAATCGGACCCTAGACAC | CAGCGAGTACATAGTCA |
| FGF21 | CTGGGGGTCTACCAAGCATA | CACCCAGGATTTGAATGACC |
| ATGL | CAACGCCACTCACATCTACG | ACCAGGTTGAAGGAGGGATG |
| HSL | CCAGGAATCCTCATTCTGGA | TGGCCAATGGATGTGAAGTA |
| GAPDH | GCACAGTCAAGGCCGAGAAT | GCCTTCTCCATGGTGGTGAA |

**3.Contents:**

Fig. S1. ^1^H NMR spectrum of **1** (CDCl_3_, 500 MHz).

Fig. S2. ^13^C NMR spectrum of **1** (CDCl_3_, 125 MHz).

Fig. S3. ^1^H NMR spectrum of **2** (CDCl_3_, 500 MHz).

Fig. S4. ^13^C NMR spectrum of **2** (CDCl_3_, 125 MHz).


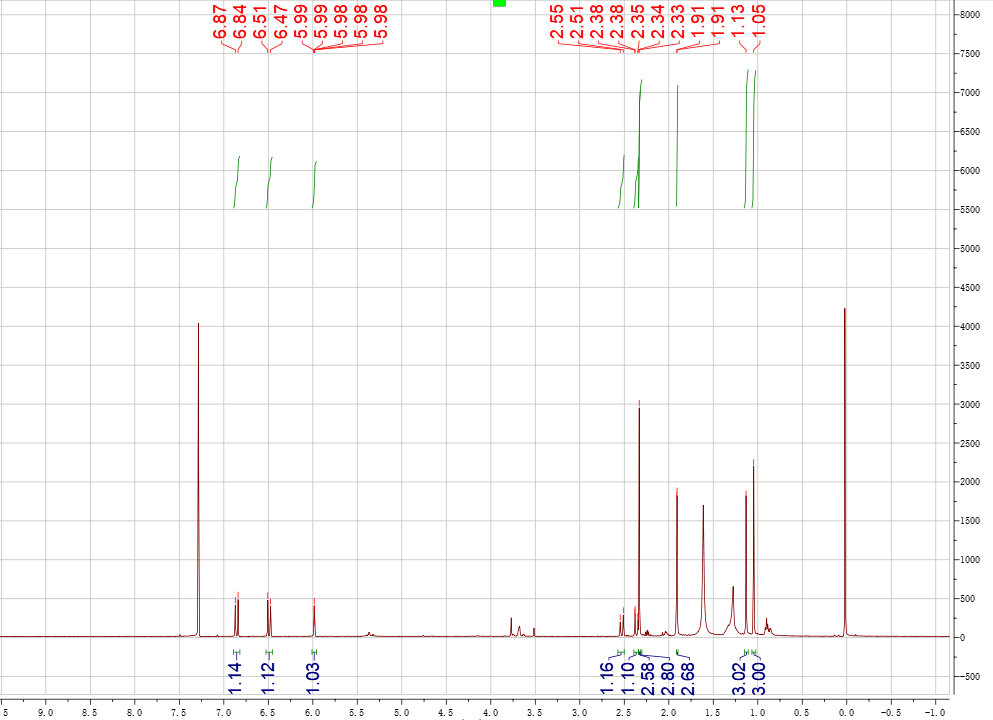


Fig. S1. ^1^H NMR spectrum of **1** (CDCl_3_, 500 MHz).


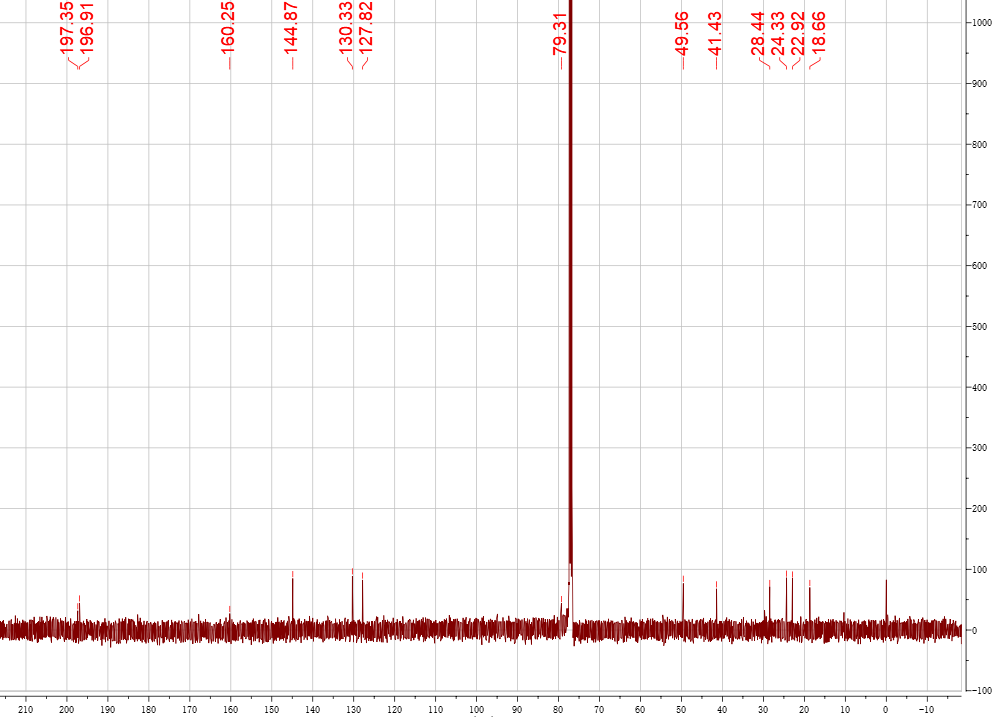


Fig. S2. ^13^C NMR spectrum of **1** (CDCl_3_, 125 MHz).


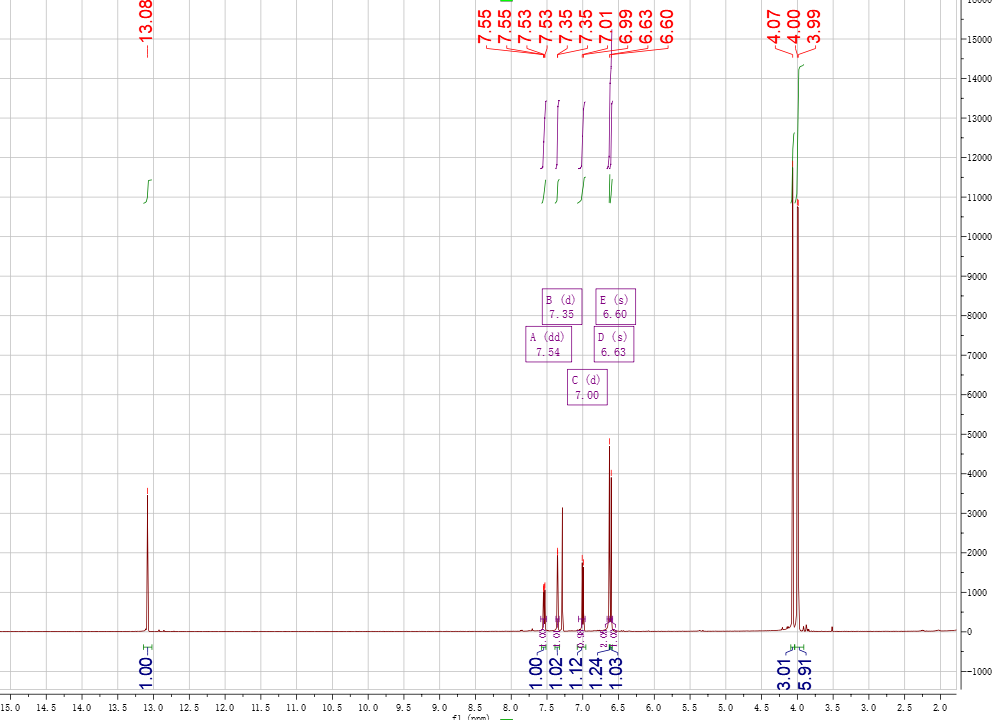


Fig. S3. ^1^H NMR spectrum of **2** (CDCl_3_, 500 MHz).


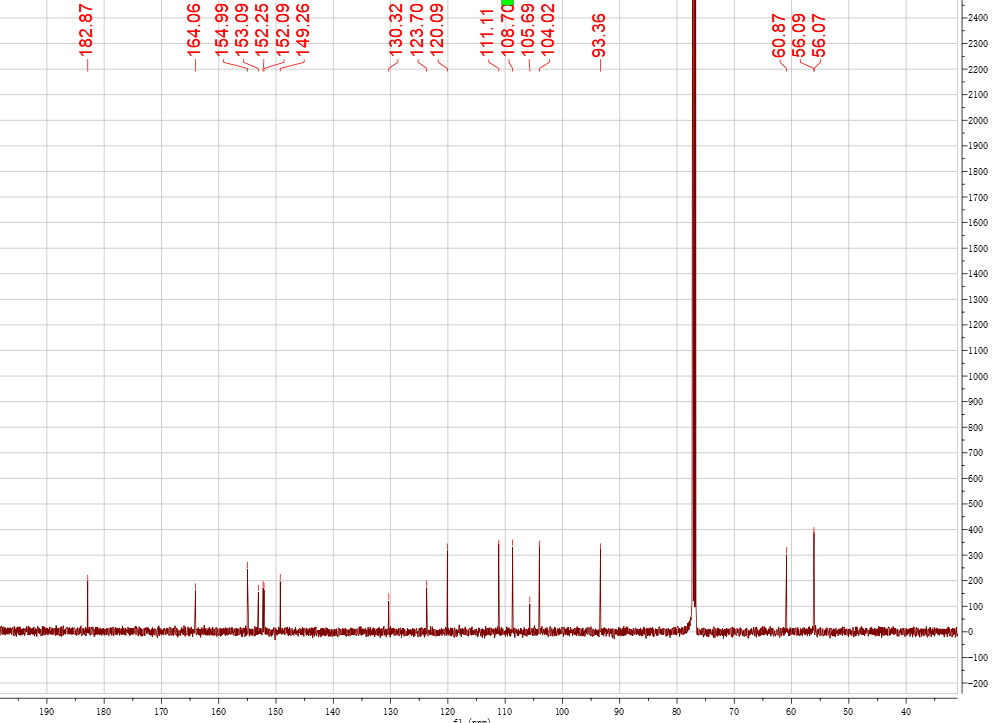


Fig. S4. ^13^C NMR spectrum of **2** (CDCl_3_, 125 MHz).
